# From “Einfühlung” to empathy: exploring the relationship between aesthetic and interpersonal experience

**DOI:** 10.1007/s10339-018-0861-x

**Published:** 2018-05-15

**Authors:** Joanna Ganczarek, Thomas Hünefeldt, Marta Olivetti Belardinelli

**Affiliations:** 1 Neurocognitive Psychology Lab, Pedagogical University of Cracow, ul. Podchorazych 2, 30-084 Cracow, Poland; 2 ECONA – Interuniversity Center for Research on Cognitive Processing in Natural and Artificial Systems, Sapienza University of Rome, Via dei Marsi 78, 00185 Rome, Italy; 3 Department of Psychology, Sapienza University of Rome, Via dei Marsi 78, 00185 Rome, Italy

**Keywords:** Einfühlung, Empathy, Aesthetics, Interpersonal experience

## Abstract

Is there a relationship between aesthetic and interpersonal experience? This question is motivated not only by the fact that historically experiences of both kinds have often been accounted for in terms of “empathy”, the English translation of the German term “Einfühlung”, but also by the fact that some contemporary theories refer to mechanisms underlying both aesthetic and interpersonal experience. In this Editorial introducing the special section titled “From ‘Einfühlung’ to empathy: exploring the relationship between aesthetic and interpersonal experience”, we briefly sketch these two motivations and the relationship between the different mechanisms that have been associated with both aesthetic and interpersonal experience.

## Introduction

Is there a relationship between aesthetic and interpersonal experience? Historically, this question is motivated by the fact that experiences of both types have been accounted for in terms of the German notion of “Einfühlung”, which Edward Titchener (1909) and James Ward (cf. Lanzoni 2012) translated as “empathy”, thus introducing the latter term into the English language. Accordingly, it is possible to distinguish between aesthetic and interpersonal “empathy” in English in much the same way as it is possible to distinguish between aesthetic and interpersonal “Einfühlung” in German, thereby suggesting a common psychological mechanism supposed to underlie both aesthetic and interpersonal “empathy”. The notion of “Einfühlung” was theoretically developed in nineteenth- and early twentieth-century German aesthetics (cf. Curtis and Elliott 2014 for a historical overview), especially by Robert Vischer (1873) and Theodor Lipps (1903, 1906). The term “Einfühlung” literally means “feeling into” and refers to an act of projecting oneself into another body or environment, i.e.—in Vischer’s (1873, p. 7) terms—to an imaginary bodily “displacement” (“Versetzung”) of oneself into another body or environment, which is aimed at understanding how it feels to be *in* that other body or environment. In other words, it refers to some kind of imaginary bodily perspective taking, which is aimed at understanding what it would be like to be living another body or another environment. Notably, the other body or the other environment where one “feels into” needs not necessarily be physically present, but it may as well be only represented, and it may even be only imaginary. For example, by “feeling into” a painted or verbally described landscape it is supposedly possible to understand what it would be like to be in that landscape and thus to understand its particular emotional tune or “atmosphere”. Similarly, by “feeling into” a portrait, a sculpture, or a tale of a human being, it is supposedly possible to understand what it would be like to be that human being and thus to understand its particular emotion or mood. Furthermore, the other body or the other environment where one “feels into” needs not necessarily be human but may be potentially any kind of body or environment. Accordingly, it is supposedly possible to “feel into” animals, plants, or even inanimate objects, whose bodies and environments are radically different from one’s own human body and environment. Therefore, the notion of “Einfühlung” is historically closely related to panpsychist ideas. In ordinary practice, however, the act of “feeling into” is usually applied rather to bodies or environments that are more or less similar to one’s own body and environment. Accordingly, it is usually applied to other human beings, but it is also readily applied to works of art, thus giving rise to the distinction between aesthetic and interpersonal “empathy”. In fact, works of art, in general, and works of figurative art, in particular, call for the act of “feeling into” another body or another environment for two main reasons: (1) all works of art are human artefacts, i.e. they have been produced by other human beings living in other historical, cultural, and personal environments, and (2) works of figurative art represent bodies or environments, and in particular often human beings or human environments. Aesthetic and interpersonal “empathy” therefore differs mainly in that interpersonal “empathy” concerns other human beings, whereas aesthetic “empathy” concerns human artefacts, especially those representing human beings or human environments.

This brief introduction into the notion of “Einfühlung” highlights several essential aspects of the psychological mechanism that supposedly underlies both aesthetic and interpersonal “empathy”. In particular, it evidences (1) the fundamental role of perspective taking, (2) the essential role of embodiment and bodily situatedness in perspective taking, and (3) the essential role of the affective, and more precisely qualitative (i.e. qualia-like), effects of such bodily perspective taking. To further illustrate the psychological mechanism that is thus supposed to be involved in both aesthetic and interpersonal “empathy”, it is helpful to consider Robert Vischer’s (1873) first theoretical account of the notion of “Einfühlung”. For Vischer, in fact, “Einfühlung” is only one of a whole set of different kinds of affective responses to objects (see Table 1). In particular, Vischer distinguished “Einfühlung”, or “feeling into”, from two other kinds of “feeling” with respect to an object, which do not involve the imaginary bodily perspective taking, or bodily “projection” *into* an object, that is characteristic of “Einfühlung”. These two other forms are “Zufühlung”, i.e. the “feeling towards” the sensory properties of an object (e.g. its brightness and colour), and “Nachfühlung”, i.e. the “feeling along” the motor properties of an object (e.g. its actual or potential movement). Notably, corresponding to these two different kinds of “feeling” which do not involve imaginary bodily perspective taking, Vischer distinguished two different kinds of “Einfühlung”, i.e. two different kinds of “feeling” which result from imaginary bodily perspective taking, namely “sensory empathy” (“sensitive Einfühlung”), i.e. the “feeling into” the sensory properties of an object, and “motor empathy” (“motorische Einfühlung”), i.e. the “feeling into” the motor properties of an object. Furthermore, corresponding to the resulting four different kinds of “feeling” with respect to an object, Vischer distinguished four different kinds of “sensation” (“Empfindung”), namely “Zuempfindung”, “Nachempfindung”, and sensory and motor “Einempfindung”. For Vischer, “feeling” differs from “sensation” in that it is less “primitive” and “more objective”, i.e. it is a somewhat more elaborate mental state that involves being aware of others having similar feelings as oneself. Thus, Vischer’s (1873) first theoretical account of the notion of “Einfühlung” does not only illustrate the fundamental role of imaginary bodily perspective taking in “empathy”, but it also illustrates two further features that are somewhat in contrast with some contemporary notions of “empathy”. In fact, it implies that “empathy” needs not necessarily be related to the motor properties of an object and it implies that “empathy” involves the awareness that there are others who have similar mental states as oneself.

**Table 1:** Different kinds of affective responses to objects according to Vischer (1873)

Affective response	Concerning an object’s sensory properties	Concerning an object’s motor properties
*Not involving perspective taking*		
Without awareness of others having similar feelings as oneself	*Zuempfindung* (sensing towards)	*Nachempfindung* (sensing along)
With awareness of others having similar feelings as oneself	*Zufühlung* (feeling towards)	*Nachfühlung* (feeling along)
*Involving perspective taking*		
Without awareness of others having similar feelings as oneself	*sensitive Einempfindung* (sensing into)	*motorische Einempfindung* (sensing into)
With awareness of others having similar feelings as oneself	*sensitive Einfühlung* (feeling into) = “sensory empathy”	*motorische Einfühlung* (feeling into) = “motor empathy”

Nowadays, the historical and conceptual roots of the concept of “empathy” and the related historical account of the relationship between aesthetic and interpersonal experience have got partially out of sight. Yet, there are also some contemporary proposals that refer to a common, fundamental mechanism underlying both aesthetic and interpersonal experience. Vittorio Gallese’s (2001) shared manifold hypothesis is one of the most important approaches that explicitly deal with the issue in question. Gallese proposes that our brains are hard-wired not only for understanding other people’s emotions, actions and intentions but also for understanding artworks. While the mirror neurons system is supposed to be the neural base allowing such understanding, embodied simulation is supposed to be the psychological mechanism responsible for it. Embodied simulation entails activation of internal representations of body states that correspond to the observed body states. This mirroring mechanism gives rise to the “as if” experience, i.e. simulation of being involved in a similar emotion or action. In the interpersonal context, Gallese proposes that embodied simulation is a basic mechanism for social identification and intentional attunement (Gallese 2009). In the aesthetic context, it is considered a basic mechanism for experiencing the content (i.e. depicted actions, emotions and sensations) and form (i.e. visible traces of artist’s creative gestures) of artworks. Besides mirror neurons, Gallese argues that also a second class of neurons, namely canonical neurons, could be crucial for embodied simulation in response to objects depicted in artworks. Canonical neurons, contrary to mirror neurons, are not active during action observation but are active when looking at objects. Gallese proposes that canonical neurons allow viewers to simulate a possible interaction with observed objects. Importantly, despite the fact that these two classes of neurons are activated in different situations, both of them underlie the same mechanism, i.e. embodied simulation. Even more importantly, while the author admits that other factors influence aesthetic as well as interpersonal experience (e.g. context, familiarity), he argues that embodied simulation is the common basic mechanism.

The idea of embodied simulation thus differs from “Einfühlung” insofar as (1) it doesn’t require imaginary bodily perspective taking, i.e. the imaginary bodily “displacement” *into* an object and, (2) it is necessarily related to the motor properties of an object (e.g. its real or potential motion, its affordances, and possibly its being an artefact) but not to its sensory properties (e.g. its brightness or colour). What instead connects the notion of embodied simulation with “Einfühlung” is the stress on embodiment in terms of activation of associated bodily states and their role in perceivers’ affective experiences.

Another proposal which makes a direct connection between interpersonal and aesthetic experience is the imitative decoding theory developed by Vezio Ruggieri (1986, 1997, 2001). The theory was initially based on studies on imitation of facial expressions, but soon it was also applied to the domain of art. The shift from interpersonal to aesthetic experience was accomplished through the notion of imitation that is believed to be a mechanism underlying both types of experiences. The theory states that perception and decoding of external forms of objects or people involves an imitation—via muscular tension—of the lines of tension that these forms depict or imply. This imitation represents an even stronger claim of embodiment with respect to Gallese’s account of embodied simulation. Whereas embodied simulation relied on brain-based representations of body states, Ruggieri’s account of imitation relies on actual modifications of muscular tension. These modifications of muscular tension cause affective experiences which influence the perception of objects. In other words, viewers feel certain bodily sensations which are attributed to the perceived object. Moreover, Ruggieri proposes that besides the imitative mechanism existing in both interpersonal and aesthetic situations, these two contexts share one more aspect. He argues that there is an important precondition necessary for a successful contact with a person or an artwork. This precondition relies on a preliminary attitude characterised by an optimal level of basic muscular tension that one must assume in order to come into contact with an external object and imitate it. Once this initial precondition is satisfied, the imitative decoding can take place. Imitative decoding thus differs from “Einfühlung” in that, similarly to Gallese’s theory, no particular imaginary bodily “displacement” is necessary, but it is enough to “resonate” with an object. What connects imitative decoding with “Einfühlung” is the focus on the bodily experience of perceivers and the link between bodily muscular response and affective experiences.

Other approaches referring to mechanisms underlying both aesthetic and interpersonal experience contribute further to the issue in question. For example, Van de Cruys and Wagemans (2011) see predictive coding as a common mechanism allowing synchronisation in aesthetic and interpersonal situations. In their view, perception of artworks and other people is linked with expectations about the incoming information which leads to generation of predictive models of, for example, intentions. If the models meet reality, i.e. if a viewer understands someone else’s intention, empathic attunement can occur causing affectively coloured interaction with an artistic or social stimulus. In a similar vein, Leder et al. (2004) propose that the aesthetic and interpersonal experience converge when viewers elaborate on an artist’s intentions. Other authors stress that emotional sharing might be important in both contexts: experiencing sadness or fear with other viewers is similar to sharing emotions in everyday life (Egloff 2017). Others again argue that the trait empathy which facilitates interpersonal experiences, might also facilitate the experience of emotions in art (Gerger et al. 2017) and in particular the experience of negative emotions in art (Menninghaus et al. 2017).

Considering these views on the relationship between aesthetic and interpersonal experience, our aim was to stimulate an interdisciplinary debate and provide a new perspective on contemporary accounts of the relationship between these two types of experiences. The papers collected in this special section address the relationship between aesthetic and interpersonal experience from a variety of different angles, demonstrating the impact and versatility of both processes, on the one hand, and their complexity, on the other. The contributing authors propose multiple distinct theoretical approaches such as philosophy, developmental psychology, psychophysiology, experimental aesthetics and service design. Within these contexts, they describe different mechanisms that might be found in both interpersonal and aesthetic experience. Gerger et al. (2018) refer to simulation and its basis in neural mirroring, following Freedberg and Gallese’s account of embodied aesthetic experience (Freedberg and Gallese 2007). They propose that a specific aspect of empathic ability, i.e. emotional contagion, might have a role in aesthetic experience. Stamatopoulou (2018) argues from a developmental perspective that both aesthetic and interpersonal experiences share the moment of sensorimotor synchrony and spatiotemporal proximity which allow more advanced processes of embodied perception and imagination to take place. Taken together these processes form the act of expressive symbolic communication with another human being or a work of art. Esrock (2018) argues that aesthetic and interpersonal experiences meet in a particular process of projection called transomatization. Transomatization occurs when viewers reinterpret a component of their own bodies to serve as a correlate, for something outside of the self, specifically, some quality of an art work or its production. Importantly, the ability for transomatization is based on early experiences of interpersonal engagement and on development of intersubjective experiences throughout life. Brinck (2018) proposes that aesthetic and interpersonal experiences meet in the process of entrainment, i.e. in the tendency of physical and biological systems to synchronise their actions and movements. This motor synchrony allows viewers to empathise with artworks or other people. It also allows them to experience the affective qualities associated with the movements. Finally, Xenakis (2018) argues that the aesthetic experience can be embedded in an interpersonal experience allowing construction of meaning, evaluation and achievement of particular goals.

Already this brief presentation of the mechanisms referred to by the authors contributing to this special section shows that their claims regarding the relationship between aesthetic and interpersonal experience differ on many levels. They provide various answers to the questions regarding its roots, its character and its strength. Thus, the papers presented in this special section invite reflection upon various aspects of the relationship between aesthetic and interpersonal experience. They present an interesting and diversified picture that might inspire new experimental studies and theories.
